# Qualitative and Quantitative Evaluation of the Make Safe Happen App: Mobile Technology–Based Safety Behavior Change Intervention for Parents

**DOI:** 10.2196/12022

**Published:** 2019-03-14

**Authors:** Kristin J Roberts, Rebecca J McAdams, Orie V Kristel, Alison M Szymanski, Lara B McKenzie

**Affiliations:** 1 Center for Injury Research and Policy The Research Institute Nationwide Children’s Hospital Columbus, OH United States; 2 Illuminology Columbus, OH United States; 3 Department of Pediatrics College of Medicine The Ohio State University Columbus, OH United States; 4 Division of Epidemiology College of Public Health The Ohio State University Columbus, OH United States

**Keywords:** smartphone, mobile phone, mobile app, parents, focus groups, technology

## Abstract

**Background:**

Nearly half of the unintentional injuries in children happen in and around the home; many of these injuries are preventable. Providing parents and caregivers with proper injury prevention information that is easily accessible may help them make their homes safer for children.

**Objective:**

The aim of this study was to evaluate parental injury prevention awareness and home safety behaviors, motivations for and challenges to taking injury prevention and safety actions for parents as well as user experience following the use of the Make Safe Happen mobile app.

**Methods:**

A total of 40 parents with children aged 0-12 years living in Columbus, Ohio, participated in 1 of 5 focus group discussions following the completion of (1) a pretest survey, (2) use of the Make Safe Happen app, and (3) a posttest survey.

**Results:**

Following the use of the Make Safe Happen app, parents reported a significant increase in injury prevention awareness and completed 45% more home *safety behaviors* in and around their homes. Nearly all of the parents felt the app provided them with the information needed to make their home safer for their children; the great majority of parents intended to make such changes in the future.

**Conclusions:**

The combination of qualitative and quantitative data collection allowed for rich data capture and provided a deeper understanding of parents’ safety knowledge, behaviors, app use, and decision making regarding child injury prevention in and around the home. The Make Safe Happen app provides the information and motivation parents and caregivers need to help them take steps to prevent child injuries that may occur in and around their homes.

## Introduction

### Injury Prevention

Unintentional injuries, such as injuries caused by burns, falls, drowning, poisoning, and motor vehicles, are the leading cause of death among children aged 1 to 19 years [1], resulting in over 9000 deaths and more than 9 million emergency department visits annually [2]. Approximately one-half of unintentional injuries in children occur in and around the home [3]. Fortunately, parents can reduce the risk of these injuries by removing hazards from their home, consistently practicing safe behavior (eg, storing medicines and household cleaners in locked cabinets), and by properly installing and regularly using safety products such as smoke alarms, carbon monoxide detectors, stair gates, cabinet locks and latches, and television (TV) and furniture anchors [4,5].

Parents and caregivers play a critical role in the prevention of home injuries, yet barriers include locating credible injury prevention information and identifying home hazards [6] as well as obtaining and properly installing safety devices in the home [7,8]. Successful preventive strategies rely on parental education and behavior change as well as environmental modifications [8-10]. Previous research suggests that the delivery of injury prevention information should coincide with the appropriate ages and developmental stages of the child to be most effective [11]. Offering home safety information on multiple topics in conjunction with the ability to easily acquire recommended safety devices may provide more efficient, wide-reaching means to reduce these potential barriers and encourage parental behavior change.

Mobile technologies, specifically apps (on mobile phones) provide a means to deliver efficient and cost-effective health information. It is estimated that nearly 80% of Americans own a mobile phone [12], and adults spend nearly 2 hours each day using mobile apps on these devices [13]. Although mobile technologies have been associated with behavior change in the areas of public health [14,15], few have been created to prevent unintentional injuries and even fewer have been systematically and rigorously evaluated [16-19]. Thus, there is a need for evaluating the effectiveness of a wide-reaching injury prevention mobile app.

### The Make Safe Happen App

The Make Safe Happen app was created to help parents and caregivers with young children (aged 0 through 12 years) make their homes safer by helping them to identify injury hazards in and around their home. The app was developed by the safety experts in the Center for Injury Research and Policy at Nationwide Children’s Hospital in partnership with Nationwide and allows users to tailor safety information by their child’s age and by features of their home. The authors sought to examine if and how parents would use an injury prevention mobile app (the Make Safe Happen app) to learn how to take safety actions in their home and adopt behaviors that could prevent unintentional injuries [20].

We conducted a series of *enhanced focus groups* to explore the following themes: (1) injury prevention awareness and home safety behaviors, (2) Make Safe Happen app user experience, and (3) motivation for taking injury prevention or safety actions and challenges to accomplishing home safety as perceived by parents with children aged 12 years and younger. Understanding how parents and caregivers used the *Make Safe Happen* app was thought to have important implications for confirming the efficacy of a potentially broad-reaching injury-prevention, behavior change intervention.

## Methods

### Study Design

We conducted 5 focus groups with parents of children aged 0 through 12 years in Columbus, Ohio, to explore attitudes and actions around home safety as well as their experience using the Make Safe Happen app. These focus group discussions are referred to as enhanced focus groups throughout this study because of the additional participant requirements (ie, pretest and posttest survey, app utilization, and focus group discussions). Enhanced focus groups allowed for both qualitative and quantitative data collection to occur for each participant. Before the focus group, parents completed a Web-based pretest and posttest survey and downloaded and used the Make Safe Happen app for 7 to 10 days. Parents completed a Web-based consent before beginning the pretest and a written consent before the focus group discussion. Following the Make Safe Happen app download, participants accepted the app’s terms and conditions. Participants were assigned a unique identification (participant ID) number which allowed for linking of the participant’s pretest, posttest, and app usage data. Participants could remove their participant ID following the focus group discussion. If the participant refused to enter the participant ID or removed the ID before completing all parts of the study, they were deemed ineligible. App utilization and analytic data for each participant were collected using Google Analytics (GA), a free Web-based analytic platform. Similar to traditional focus groups, participants were engaged in a discussion facilitated by a moderator. The focus group discussions were a part of a larger study to evaluate the effectiveness of the Make Safe Happen app on safety knowledge, actions, and behavioral intentions [20]. The study was approved by the Research Institute at Nationwide Children’s Hospital Institutional Review Board.

### Recruitment

Participants for this study were recruited by a market research firm, using its panel of individuals who had previously opted in to help with research studies. After identifying panelists that may be eligible, the market research firm screened participants for eligibility over the phone. To be eligible, participants needed to be the parent or legal guardian of at least one child aged 0 through 12 years who lived with them most of the time. The child age range was selected based on the available age categories within the Make Safe Happen app, which allows users to filter child injury prevention recommendations and content for children aged 0 through 12 years. To meet study eligibility criteria, participants had to be ≥18 years, comfortable answering questions in English, own a smart phone, and willing to download and use the Make Safe Happen app for 7 to 10 days. If parents had previously downloaded or used the Make Safe Happen app, they were not eligible for participation. Participants reflected a mix of genders, races, and ethnicities.

### Pre- and Posttest Survey

Following telephone recruitment and screening for eligibility, eligible participants were emailed a unique pretest survey link containing a Web-based consent form and questions regarding their safety behaviors, safety knowledge, safety device acquisition and use, and behavioral intentions to adopt future safety actions.

### The Make Safe Happen App, Actions, and Google Analytics Linkage

**Figure 1:**
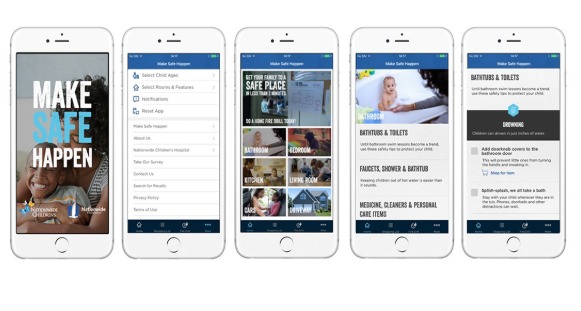
Make Safe Happen Mobile App.

Following completion of the pretest survey, participants were asked to download and use the Make Safe Happen app. General features of the app include the ability for users to tailor safety information by their child’s age (ie, 0 to 11 months, 12 to 23 months, 2 to 4 years, 5 to 9 years, and 10 to 12 years) and customize their experience by selecting features of their home (ie, standard rooms, garage, basement, stairs, and hallways). The Make Safe Happen app allows users to identify injury hazards with customized room-by-room checklists, links to purchase safety products best suited to the features of their homes via a Web-based global retailer, create shopping lists, and set reminders to encourage consistent safety behaviors. Within each room, users can select injury prevention topics; once clicked, the user will view checklists containing prevention tips and tasks for each injury prevention topic (Figure 1). Injury prevention tasks include the option to add a safety product to their shopping list, shop for products (linking the user directly to the Amazon search for that product), and the ability to add reminders to the calendar (including reminders to change batteries and test devices such as smoke detectors and carbon monoxide alarms). The Make Safe Happen app was available for free download from the Google Play store or the Apple App store. Participants downloaded the Make Safe Happen app version 2.2.0.

Upon completion of the pretest survey, participants received a unique study app identification (app ID) number and instructions via email to download the Make Safe Happen app onto their mobile phone. Participants entered the app ID, a 6-digit alphanumeric code, into the Make Safe Happen app so their actions in the app could be linked to their pretest and posttest survey data. After a period of 7 to 10 days of app use, participants were emailed a unique posttest survey link. Participants’ app usage was collected by using GA. When a participant checked an item off in the app, a data point linked to the participant ID was recorded in GA. The participant ID also allowed for data collected from nonstudy users (those without an app ID) to be separated from the study participant app analytics.

### Focus Group Discussion Themes

Participants who completed the pretest, app download, and posttest survey were invited to attend 1 of the 5 in-person focus group discussions. A total of 5, 90-min in-person focus group discussions were completed during October 2016 in Columbus, Ohio. Groups of 8 parents were organized according to the age of the index child (2 groups for parents with children aged <2 years and 1 group for parents with children aged 2 to 4 years, 5 to 9 years, and 10 to 12 years). For participants with multiple children, the index child was assigned to the group that contained the least number of parents at enrollment; these parents were asked to only discuss the index child during the focus group discussions. While waiting for the focus group discussions to begin, they completed a brief survey collecting the participants’ *home safety* and *app experience rating*. Data reported are for participants that completed all parts of the study.

A professional focus group moderator (AS) led each session through focused conversation, using a discussion guide developed by the study team to ensure critical questions and probes of interest were discussed. The research team observed the focus group discussions through a one-way mirror. Discussions were audio-taped, transcribed by a professional transcription service, and proofed for accuracy by the research team and moderator. At the conclusion of the focus group discussions, participants received a Make Safe Happen–branded gift bag containing home safety information. Participants were paid US $150 to thank them for their time. Results from the focus group discussions were paired with corresponding data collected from the pretest and posttest surveys, user experience questions, and GA and presented accordingly under each thematic subsection.

### Outcomes and Measures

The enhanced focus group design aimed to explore parents’ (1) injury prevention awareness and home safety behaviors; (2) Make Safe Happen app user experience; and (3) motivation for taking injury prevention or safety actions as well as the challenges that may inhibit these safety behaviors.

#### Injury Prevention Awareness and Home Safety Behaviors

Injury prevention awareness was measured by responses to 17 multiple choice questions asked in each of the pretest and posttest surveys based on content delivered in the Make Safe Happen app. One point was given for each correct answer, and the total of correct answers was summed and divided by the total number of questions (17) for each participant to calculate the *total safety knowledge score*. The *mean total safety knowledge score* for all participants was derived by calculating the mean of the *total safety knowledge score*.

Home safety behaviors were determined via responses to questions on both the pretest and posttest surveys that measured the participant’s *repeated safety behaviors*, which are safety behaviors that are recommended to be done repeatedly such as locking up medications, and safety behaviors, which are behaviors that are typically completed once such as installing a smoke alarm. The frequencies of the participants’ *repeated safety behaviors* and *safety behaviors* were measured by responses to 14 multiple choice questions and 15 multiple choice questions, respectively. A *home safety rating* was collected during a written exercise before the start of the focus group discussions and was measured by a 5-point Likert scale, where 1 indicated *Everything is childproofed*, 3 indicated *Safe, but room for improvement*, and 5 indicated *Nothing is childproofed*.

#### Make Safe Happen App Participant Usage and Actions

From September 29, 2016, to October 13, 2016, GA data regarding participants’ *sessions*, defined as the number of times the app was opened and at least 1 or more action was taken within the app as well as the number of app screens viewed, were collected. In addition, data of completed *app safety actions*, actions completed within the app such as *checking off* items, creating calendar reminders, or adding safety devices to a shopping list, were collected. GA provides individual and aggregate data for the total number of app sessions, screens viewed, the average number of screens viewed per session, and the average duration of time of each session for the focus group participants. GA does not generate the total amount of time spent on the app per individual. Each parent’s *app experience rating* was collected before the focus group on the written survey and was measured by rating the Make Safe Happen app on a scale of 1 (positive) to 5 (negative). Using a Likert scale where 1 indicates dislike and 5 indicates liked a lot, parents provided a *rating of features* for the room-specific checklists and the ability to customize the app based on rooms and features of their home.

#### Motivation for Prevention and Challenges to Home Safety

During the posttest survey, parents were asked to select the safety tasks that they had completed in and around their home as well as the reasons why they had not made other changes to their home during the past week. Before the start of the focus groups, parents completed a brief one-page app experience survey which asked them to describe the emotions and feelings they had regarding their app experience as well as open-ended questions regarding the features they liked the most and the least.

### Data Analysis

Survey response data were analyzed by using SPSS statistical software 24.0 (SPSS Inc) and SAS Enterprise Guide version 7.1 (SAS Institute, Inc). A thematic analysis was conducted by a process similar to that recommended by Braun and Clarke [21]. AS and OK used the focus group transcripts to identify and analyze important themes. Transcripts were created from audio recordings of the focus group discussions. Members of the study team reviewed the transcripts beginning at the section of each transcript corresponding to the questions asked by the moderator. Initial codes were

created based on the parents’ discussion for each question asked by the moderator. Similar and dissimilar responses were grouped, unique or different comments were noted, and patterns addressing the key questions of interest were summarized to extract important themes. These themes were refined and reorganized after review, keeping in mind the key objectives of the research. Any disagreements were resolved via discussion. The results are presented under each respective section: (1) injury prevention awareness and home safety behaviors; (2) Make Safe Happen app user experience; and (3) motivation for prevention and challenges to home safety.

Pretest and posttest difference of the *mean total safety knowledge score* was analyzed with the chi-square test using alpha=.05. Statistical methods were not applied to *all repeated safety behaviors* and *all safety behaviors* because of small frequencies that lacked statistical robustness. This study was approved by the Institutional Review Board at the Nationwide Children’s Hospital.

## Results

### Overview

Participants who completed both the pretest and posttest surveys and downloaded and used the app were invited to attend 1 of the 5 focus group discussions (N=49). A total of 9 participants were dismissed immediately before the focus group discussions to maintain small group sizes. Dismissal was determined based on the time of arrival, and the first 8 participants to arrive were invited into the discussions. The remaining participants were paid before dismissal. A total of 40 parents participated in 1 of 5 focus groups consisting of 8 parents within each group with 58% (23/40) mothers and 43% (17/40) fathers. Parents were, on average, aged 35.8 (SD 6.0) years, mostly white, non-Hispanic (65%, 26/40), members of a 2-parent household (75%, 30/40), educated (Bachelor’s degree or more; 75%, 30/40), and employed full time (65%, 26/40). Most parents reported owning their own home (68%, 27/40) and living in their home for ≥5 years (35%, 14/40). Parents had an annual household income of ≥US $80,000 (51%, 20/39) and reported feeling they are *meeting ends easily or very easily* with their income (63%, 25/40; Table 1).

### Injury Prevention Awareness and Home Safety Behaviors

#### Pretest and Posttest Survey

Following app use, the *mean safety knowledge score* significantly increased from 63% (10.7/17) at pretest to 81% (13.7/17) at posttest (*P*<.001). The percent of participants who completed all repeated safety behaviors increased by 20.0%, from 48% (19/40) at pretest to 68% (27/40) at posttest, and the percent of participants who completed all safety behaviors had a larger increase of 45%, from pretest (15%, 6/40) to posttest (50% (20/40); Table 2). On the posttest survey, one parent did not use the app within the study period, and over 60% of parents (62%, 24/39) reported that they were already following *safety behavior*.

#### Focus Group Discussions

During the focus groups, parents reported that they could be doing more to prevent injuries in their homes and that they became more aware of the different ways they could make their homes safer, following app use. On average, participants gave their homes a *home safety rating* of 2.7 (range 2.5-2.9), just better than *Safe, but room for improvement*. Parents discussed that they had taken several safety measures such as installing outlet covers, doorknob covers, baby gates, cabinet locks, window stops, and blind cord shorteners; securing cleaning products; securing furniture to the wall; and checking fire alarm batteries and believed these actions had helped prevent injuries. All parent participants reported that they were motivated to install safety devices; however, the type of safety devices mentioned for installation differed by child age.

During the focus group discussions, parents of younger children (aged 0 to 2 years) were particularly motivated to install, test, and/or purchase safety devices such as TV and furniture anchors, carbon monoxide alarms, oven locks, corner guards, and window locks. Parents of children aged over 2 years were motivated to improve home fire safety and were especially interested in testing, installing, and purchasing products such as smoke alarms, fire extinguishers, and fire escape ladders, as well as preparing for a home fire by practicing a home fire drill using the Make Safe Happen app:

I had added the tethers to all the furniture when my seven-year-old and my five-year-old were young, but...now we have a nine-month-old, and we had to purchase other furniture...I thought I was golden and all that, but there was a good reminder...and there was one TV that we didn’t have attached to the wall...I didn’t even know there was a water temperature thing you could do. I’d never heard of that before, either. So, I need to look more into that.

Parent of a child aged 0 to 2 years

We only had our really old fire extinguisher, and it’s…in the garage…this made me think, like “Oh, we should probably have one out there and one in the house,” and we haven’t checked it…It probably doesn’t even work…I hadn’t thought about that at all, and I’m pretty safety-conscious, so that’s one thing that’s on the list to get.

Parent of a child aged 10 to 12 years

**Table 1:** Demographic characteristics of enhanced focus group participants.

Demographic characteristics		Focus group participants (n=40)	Parents of children <2 years (n=16)	Parents of children 2-4 years (n=8)	Parents of children 5-9 years (n=8)	Parents of children 10-12 years (n=8)
Age (years), mean (SD)		36 (6)	34 (5)	32 (3)	37 (7)	41 (60)
Number of children <18 years in household, mean (SD)		2 (1)	2 (1)	2 (1)	2 (1)	3 (1)
**Gender, n (%)^a^**						
	Fathers	17 (43)	7 (44)	3 (38)	4 (50)	3 (38)
	Mothers	23 (58)	9 (56)	5 (63)	4 (50)	5 (63)
**Race or ethnicity, n (%)**						
	White, non-Hispanic	26 (65)	12 (75)	5 (63)	4 (50)	5 (63)
	Black, non-Hispanic	10 (25)	2 (13)	2 (25)	3 (38)	3 (38)
	Other	4 (10)	2 (13)	1 (13)	1 (13)	0 (0)
**Education, n (%)**						
	≤High school or General Education Development	1 (3)	0 (0)	1 (13)	0 (0)	0 (0)
	Some college	9 (23)	4 (25)	3 (38)	1 (13)	1 (13)
	≥Bachelor's degree	30 (75)	12 (75)	4 (50)	7 (88)	7 (88)
**Employment, n (%)**						
	Full Time	26 (65)	12 (75)	4 (50)	6 (75)	4 (50)
	Part-Time	5 (13)	2 (25)	1 (13)	2 (25)	0 (0)
	Stay at home parent	8 (20)	1 (6)	3 (38)	0 (0)	4 (50)
	Other	1 (3)	1 (6)	0 (0)	0 (0)	0 (0)
**Household, n (%)**						
	Two Parent	30 (75)	15 (94)	4 (50)	5 (63)	6 (75)
	Single Parent	10 (25)	1 (6)	4 (50)	3 (38)	2 (25)
**Home ownership, n (%)**						
	Own	27 (68)	12 (75)	2 (25)	6 (75)	7 (88)
	Rent	13 (33)	4 (25)	6 (75)	2 (25)	1 (13)
**Time in current household, n (%)**						
	≥5 years	14 (35)	4 (25)	2 (25)	5 (33)	3 (38)
	3-4 years	12 (30)	7 (44)	2 (25)	1 (13)	2 (25)
	1-2 years	10 (25)	3 (19)	4 (50)	1 (13)	2 (25)
	≤11 months	4 (10)	2 (13)	0 (0)	1 (13)	1 (13)
**Income^b^, n (%)**						
	<$20,000	1 (3)	0 (0)	1 (13)	0 (0)	0 (0)
	$20,000-$39,999	5 (13)	1 (6)	3 (38)	1 (13)	0 (0)
	$40,000-$59,999	8 (21)	3 (19)	0 (0)	0 (0)	5 (63)
	$60,000-$79,999	5 (13)	3 (19)	2 (25)	0 (0)	0 (0)
	≥$80,000	20 (51)	9 (56)	1 (13)	7 (88)	3 (38)
**Livability, n (%)**						
	Meet ends with difficulty or with great difficulty	3 (8)	4 (25)	1 (14)	1 (13)	0 (0)
	Just get by	12 (30)	0 (0)	4 (43)	0 (0)	5 (33)
	Meet ends easily or very easily	25 (63)	12 (75)	3 (29)	7 (88)	3 (38)

^a^Total percentage may not add to 100 because of rounding.

^b^Income missing n=1 because participant preferred not to answer.

**Table 2:** Percentage of mean safety knowledge score and of participants who completed all safety behaviors.

Study Measures	Pretest	Posttest
Mean safety knowledge score^a^	63	81
All repeated safety behaviors, n (%)	19 (48)	27 (68)
All safety behaviors, n (%)	6 (15)	20 (50)

^a^*P*<.001.

### Make Safe Happen App User Experience

#### Make Safe Happen App Participant Usage and App Safety Actions

There were 229 app sessions during the study period for the 40 participants who completed the focus group discussions. During these app sessions, 3314 screens were viewed averaging 14.5 screens per session for an average duration of 8 min and 24 seconds. Participants completed 1972 *app safety actions*. A total of 42% (17/40) of parents completed all tasks for at least 1 room in the app. Of the standard 4, the bedroom (22%, 9/40) and kitchen (20%, 8/40) were most commonly completed within the app by participants. Participants set a total of 19 calendar reminders. Testing smoke alarms had the greatest number of set reminder actions (n=7), followed by testing carbon monoxide alarms and replacing smoke alarm batteries, where n=3 for both, and 1 person reported completing the action to replace carbon monoxide alarm batteries (Textbox 1).

#### Focus Group Discussions

Most parents (73%, 29/40) reported that they had a positive experience while using the app. On average, parents gave the app an *app experience rating* of 2.1. Parents of children aged less than 2 years had a particularly positive experience, with an average *app experience rating* of 1.7, whereas the average among parents of children aged 2 years and older was 2.3. Almost all parents (93%, 37/40) indicated they felt *informed* while using the app. Other common emotions reported were positive, including feeling *encouraged* (58%, 23/40), *empowered* (40%, 16/40), and *engaged* (40%, 16/40):

Informative of things I didn’t know about but also made me feel I should push to make my home safer and be more alert.

Parent of a child aged 0 to 2 years

It was very detailed. I’m a checklist person and I love that about it! It made me thinking about things in different areas that I wouldn’t have thought of.

Parent of a child aged 0 to 2 years

I thought it was easy to use, but I really liked how it was organized by room, because it wasn’t so overwhelming.

Parent of a child aged 10 to 12 years

During the discussions, parents reported that they typically only checked off items in the app checklist after they completed the *app safety action*; *a* *pp safety actions* added to the participant’s *mental checklist* were not checked off in the Make Safe Happen app checklist and thus would not be recorded as an *app safety action* in GA. Parents liked the option to add the Poison Help Number to their contacts; however, only about one-third (14/40) of them reported adding the number to their mobile phones and only 20% (8/40) actually completed that *app safety action*. During the discussion, some parents said they already had it in their mobile phone contacts and others said they would “Google it” if they needed to call the number. In addition, parents liked the shopping list and calendar functions of the Make Safe Happen app, yet they reported that they did not use these features (Table 2). Parents reported not using the shopping features because they either do not often use their phones to shop or because they needed to discuss their purchases with their spouse or partner. Participants who reported not using the calendar feature either do not use the calendar on their mobile phone or already have a plan to regularly change their smoke alarm (or carbon monoxide alarm) batteries.

Parents provided a *rating of features* for the room-specific checklists and the ability to customize the app, both scoring means of 4.5 (liked a lot). Parents of children aged below 5 years seemed likely to keep using the app, whereas parents with children aged 5 years or above seemed less likely to continue to use the Make Safe Happen app:

It has good information...I think [I’ll] keep it around, is because I know even if you were to

have everything checked off, it’s just good to look at that list just to reaffirm it.

Parent of a child aged 0 to 2 years

I think I’ll keep it, just because as my kids get older, I can change the ages on it and it will adjust the differences that might be applicable to their age groups.

Parent of a child aged 2 to 4 years

I think once I went through all the rooms and set the calendar reminders, I think the only reason may be to keep it would be–like the fire escape plan, as the kids get older, let them practice and use the phone.

Parent of a child aged 5 to 9 years

**The Make Safe Happen app safety actions as recorded in Google Analytics.**

Index child age

- 12-23 months (n=16)
- 2-4 years (n=8)
- 5-9 years (n=8)
- 10-12 years (n=8)

Selected child age in app (study participants were able to select the ages of all their children in the app, including the index child age)

- 0-11 months (n=9)
- 12-23 months (n=10)
- 2-4 years (n=18)
- 5-9 years(n=18)
- 10-12 years (n=13)

Rooms completed

- Any room (a total of 17 participants completed all of the tasks for at least one room; n=17)
- Bathroom completed (n=5)
- Basement completed (n=3)
- Bedroom completed (n=9)
- Cars completed (n=4)
- Driveway completed (n=4)
- Fireplace completed (n=3)
- Garage completed (n=2)
- Kitchen completed (n=8)
- Laundry Room completed (n=6)
- Living Room completed (n=5)
- Playroom completed (n=1)
- Stairs & Hallways completed (n=6)
- Yard completed (n=1)

Set reminder

- Total set reminders (n=19)
- Testing smoke alarms (n=7)
- Testing carbon monoxide alarms (n=3)
- Replacing smoke alarm batteries (n=3)
- Replacing carbon monoxide alarm batteries (n=1)

Check off safety actions (n=1741)

Shop for (n=39)

Shop for shopping list (n=19)

Shop for amazon (n=14)

Social share (n=3)

Added poison help number to contacts (n=8)

Total app safety actions (n=1972)

**Table 3:** Self-reported parental barriers to take safety actions.

Barriers to taking safety actions	Statistics, n (%)^a^
Intend to make changes in near future	34 (87)
Already follow these safety recommendations	24 (62)
Do not have time to make changes	13 (33)
Do not think the information is relevant to me	10 (26)
Cannot afford safety products now	9 (23)
Did not take safety action or actions for some other reason	6 (15)
Am not ready to follow safety recommendations	4 (10)

^a^Total does not add to 100% because participants could select more than one barrier.

### Motivation for Prevention and Challenges to Home Safety

#### Pretest and Posttest Survey

On the posttest survey, one-third of the parents (33%, 13/39) reported not having enough time to make the recommended changes, whereas approximately one-quarter of parents reported not being able to afford these changes (23%, 9/39) or that they did not think the recommendations were relevant to them (26%, 10/39). However, almost 90% (87%, 34/39) of parents reported intending to make changes in the future (Table 3).

#### Focus Group Discussions

Parents described feeling overwhelmed with the amount of safety tasks included in the Make Safe Happen app.

It was overwhelming. I was like, oh my gosh, we’re failing.

Parent of a child aged 0 to 2 years

For parents of children of all ages, making the safety tasks easier would motivate them to complete injury prevention tasks. They said they would be more likely to complete these safety tasks if they had more time, reminders, easier access to safety products, less expensive products, and assistance with installation of safety devices. Parents also mentioned that some parents and guardians or family members may be another barrier to taking safety actions:

A barrier is actually...my husband...when I bring stuff up, he’s like, “Why? We didn’t have fire ladders when we were a kid, so why?” ...I begged for Christmas for in-laws to buy a fire extinguisher, and he’s like, “Why do we need one in the basement?”

Parent of a child aged 0 to 2 years

I think cost could potentially be something that would maybe put someone–not be able to do everything. Especially with the window stop sand the fire escape ladders. That’s a large expense, and if you have a lot of upstairs rooms.

Parent of a child aged 5 to 9 years

Additionally, some parents mentioned *seeing the risks of not doing* tasks or a *near miss situation* would motivate them to further take safety actions in their home:

This last week has been really cool, like where I want the windows open at night, but then I’m looking at it, like, I guess they could [fall out]...so then I was, “I guess it’s not worth the risk,” so I did close up the windows and lock ‘em, since we don’t have bars or anything in there for now...It did make me conscious.

Parent of a child aged 0 to 2 years

I never did the TV strap…I’ve never really had a close call with it, but I guess you don’t want to have a close call, because that might be it…I can lift up my TV with…one hand…I guess maybe I didn’t believe it, that it could kill a kid.

Parent of a child aged 2 to 4 years

## Discussion

### Principal Findings

This study demonstrates that an injury prevention mobile app, Make Safe Happen, can be used to improve parental safety knowledge and positively influence parental behavior by motivating parents to complete safety actions to make their home safer for their children. By using enhanced focus groups to combine qualitative and quantitative data, this study was able to deeply explore parental knowledge, behaviors, and decisions regarding child injury prevention in and around the home following the use of the Make Safe Happen app. Although previous work has highlighted successful injury prevention strategies that incorporate parental education, behavior change, and environmental modification [8-10], this methodology allowed for further exploration of each component following app use. Our findings suggest that the Make Safe Happen app may be a valuable resource that can provide parents with the knowledge and resources needed to encourage the completion of child injury prevention tasks in and around the home. To our knowledge, this study is the first to utilize enhanced focus groups to evaluate a child injury prevention app developed to help parents and caregivers keep their children safe in and around the home.

Strategies to prevent home injuries to children include increasing parental safety knowledge and environmental modification and facilitating behavior change [5]. Effective injury prevention interventions may incorporate all 3 components as increasing awareness and knowledge may facilitate behavior change including environmental modification such as the installation and proper use of safety devices. Similar to other research evaluating technologies developed to increase child injury prevention knowledge and behaviors [22,23], parents in our study reported a significant increase in injury prevention awareness measured by their *mean* *safety knowledge score* and completed 45% more home *safety behaviors*, following the use of the Make Safe Happen app. Following app use, parents stated the app content helped raise their awareness regarding the safety of their homes, the need to make their homes safer, and the ability to know the safety measures needed to improve their home safety.

Although removing hazards and installing safety devices can reduce the risk of home injury [4], parents may find it difficult to complete these tasks because these modifications often take time, money, resources, and may vary by child age and developmental stage [11,24,25]. Parents of younger children (aged 0 to 2 years, with no previous older children) may have felt more overwhelmed by the information presented because they may not have previously thought about and/or taken steps toward preventing injuries in their homes. However, these parents gave the app a more positive *app experience rating* and were most motivated to improve the safety of their home by installing safety devices.

Although we were able to view the app analytics to confirm that parents opened the app, viewed screens, and completed actions within the app, these actions do not correlate to the actions completed within the parent’s home. During the focus group discussions, parents shared that while they used the app and explored the content, they did not physically mark the action as completed in the app until the action was completed in their home. Thus, items added to a *mental checklist* would not be documented as completed in the app if they simply read and acknowledged that this task was something they wanted to do. Similarly, parents reported liking certain app features, such as adding the Poison Help Number to their contacts, even though they decided not to use the features during the study period. Following use of the app, parents stated they would be more likely to complete tasks around their home that could prevent child injuries if they had more time, more reminders, easier access, and less expensive products as well as assistance to complete these tasks. Future directions could explore opportunities to incorporate additional reminders and resources such as discounts on safety products and installation services to help motivate task completion. Although continued app use following the study is unknown, parents of children aged below 5 years expressed an interest in continued app use and reported a positive app experience, stating they felt informed, encouraged, empowered, and engaged while using the app.

### Limitations

This study has several limitations. First, our sample consisted of predominately white non-Hispanic participants, and there was no control or comparison group for this portion of the study, thus limiting the generalizability of these findings. In addition, participants were existing members of a research panel. Participants may have agreed to participate because they were active technology users or had an interest in injury prevention or research, which may have introduced self-selection bias. For the purposes of the focus groups organization, participants were asked to only discuss the child(ren) that was within that age group, although parents may have had additional children in other age groups and this additional parenting experience or knowledge may have biased some of their discussions. However, parents were asked to use the app as they naturally would if they had found it on their own. In addition, the content within the Make Safe Happen app focuses more heavily on injury prevention information relevant to younger children. Therefore, parents with only older children may have not been exposed to all injury prevention topics. Although participants were aware that their app use was being monitored, they were not reminded or prompted to use the app during the study period, which could have increased app use during this limited period of 7 to 10 days. Finally, no observed safety behaviors or long-term outcomes such as app usage were collected, thus limiting our understanding of participant app usage and safety behavior change following our study period.

Despite these limitations, this study brings to light important information regarding parental attitudes, knowledge, and behaviors regarding child injury prevention in the home. Mobile delivery is a feasible and wide-reaching option to provide home injury prevention information to parents. This methodology provides a cost-effective approach to obtain valuable, qualitative, and quantitative data regarding one’s understanding and practice before the intervention (Make Safe Happen app use), actual app usage, and detailed discussions concerning their perceived barriers and motivators for injury prevention. Future directions may explore the benefits of additional reminders, notifications, and links to resources within the app. Additional research should investigate continued app use and user engagement and motivators for the completion of home injury prevention tasks for parents with children of all ages.

### Conclusions

The combination of the qualitative and quantitative data greatly enriched our understanding of parents’ attitudes, knowledge, and behaviors regarding the use of a child injury prevention mobile app, Make Safe Happen. Following app use, parents had an increase in knowledge and self-reported home safety behaviors. Although parents had a positive app experience, barriers to home safety such as time as well as access to and cost of safety products remain challenges delaying or preventing the completion of some home safety tasks. The Make Safe Happen app may provide parents and caregivers with the information and motivation needed to help prevent child injuries in and around their homes. The enhanced focus group approach is a valuable and cost-effective method that allows for rich data capture and provides a deeper investigation into the actions, behaviors, and decision making of parents regarding child injury prevention in and around the home. Future research should evaluate long-term user engagement and behavior change for parents with children of all ages following the use of a mobile app designed for child injury prevention.

## Abbreviations

GA: Google Analytics
TV: television
